# Mauritian Endemic Medicinal Plant Extracts Induce G2/M Phase Cell Cycle Arrest and Growth Inhibition of Oesophageal Squamous Cell Carcinoma in Vitro

**Published:** 2019

**Authors:** N. Rummun, R. E. Hughes, R. Beesoo, W. W. Li, O. Aldulaimi, K. G. Macleod, T. Bahorun, N. O. Carragher, A. Kagansky, V. S. Neergheen-Bhujun

**Affiliations:** Department of Health Sciences, Faculty of Science, University of Mauritius, Réduit, 80837, Republic of Mauritius; ANDI Centre of Excellence for Biomedical and Biomaterials Research, MSIRI Building, University of Mauritius, Réduit, 80837, Republic of Mauritius; Guy Hilton Research Centre, Institute for Science and Technology in Medicine, Faculty of Medicine and Health Sciences, ST5 5BG, Keele University, UK; Cancer Research UK Edinburgh Centre, MRC Institute of Genetics and Molecular Medicine, Western General Hospital, University of Edinburgh, Edinburgh, EH4 2XU, United Kingdom; Department of Biosciences and Ocean Sciences, Faculty of Science, University of Mauritius, Réduit, 80837 , Republic of Mauritius; Centre for Genomic and Regenerative Medicine, School of Biomedicine, Far Eastern Federal University, 690091, Vladivostok, Russia

**Keywords:** Mauritian endemic, medicinal plant, oesophageal carcinoma, tumor cytotoxicity, AMPK

## Abstract

Terrestrial plants have contributed massively to the development of modern oncologic drugs. Despite the wide acceptance of Mauritian endemic flowering plants in traditional medicine, scientific evidence of their chemotherapeutic potential is lacking. This study aimed to evaluate the *in vitro *tumor cytotoxicity of leaf extracts from five Mauritian endemic medicinal plants, namely *Acalypha integrifolia *Willd (Euphorbiaceae), *Labourdonnaisia glauca *Bojer (Sapotaceae), *Dombeya acutangula *Cav. subsp. *rosea *Friedmann (Malvaceae), *Gaertnera psychotrioides *(DC.) Baker (Rubiaceae), and *Eugenia tinifolia *Lam (Myrtaceae). The cytotoxicities of the extracts were determined against six human cancer cell lines, including cervical adenocarcinoma, colorectal carcinoma, oesophageal adenocarcinoma, and oesophageal squamous cell carcinoma. The potent extracts were further investigated using cell cycle analysis and reverse phase protein array (RPPA) analysis. The antioxidant properties and polyphenolic profile of the potent extracts were also evaluated. Gas chromatography mass spectrometry (GC-MS) analyses revealed the presence of (+)-catechin and gallocatechin in *E. tinifolia *and *L. glauca*, while gallic acid was detected in *A. integrifolia*. *L. glauca*, *A. integrifolia, *and *E. tinifolia *were highly selective towards human oesophageal squamous cell carcinoma (KYSE-30) cells. *L. glauca *and *E. tinifolia *arrested KYSE- 30 cells in the G2/M phase, in a concentration-dependent manner. RPPA analysis indicated that the extracts may partly exert their tumor growth-inhibitory activity by upregulating the intracellular level of 5′AMP-activated kinase (AMPK). The findings highlight the potent antiproliferative activity of three Mauritian endemic leaf extracts against oesophageal squamous cell carcinoma and calls for further investigation into their chemotherapeutic application.

## INTRODUCTION

**Fig. 1:**
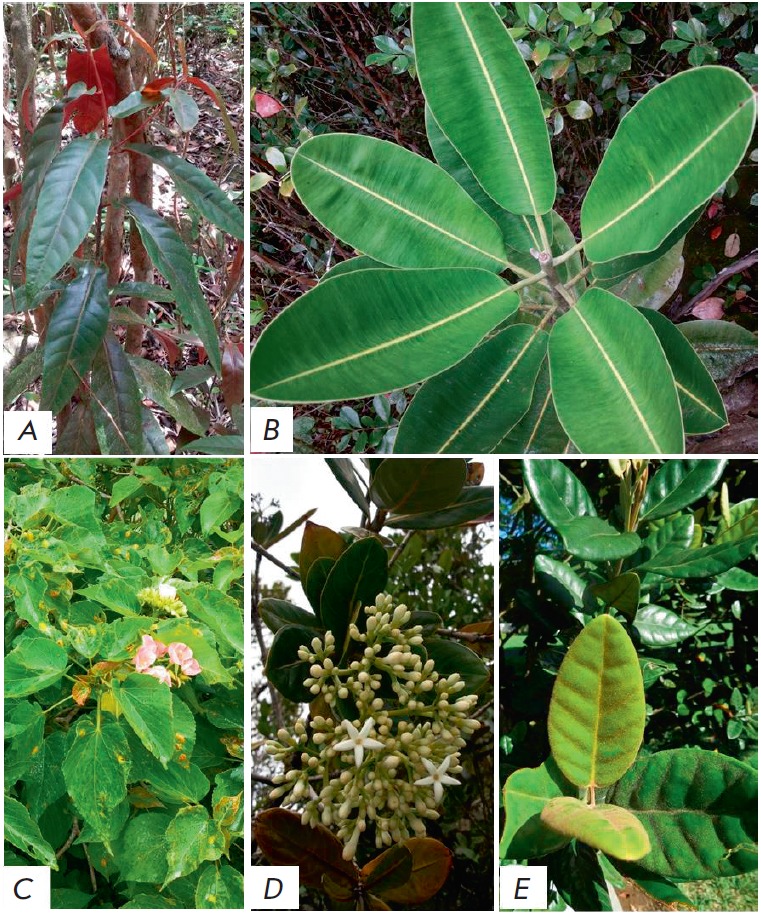
The Mauritian endemic medicinal plants under study. The Mauritius herbarium voucher specimen barcode number is given in brackets ( ). *A *– *A. integrifolia *(MAU 0016402); *B *– *L. glauca *(MAU 0016430); *C – D. acutangula *(MAU 0016638); *D *– *G. psychotrioides *(MAU 0009450); *– E. tinifolia *(MAU 0016540)

Oesophageal cancer (OC) is of increasing global concern due to poor prognosis, aggressive disease course, and a lack of efficient selective therapeutics. Oesophageal squamous cell carcinoma and oesophageal adenocarcinoma represent the sixth leading cause of cancer death worldwide and are associated with a 5-year survival of less than 15% and a median overall survival of less than a year [1]. Current treatment options rely primarily on broad-spectrum cytotoxic chemotherapeutics such as cisplatin, fluorouracil, and paclitaxel, which are associated with toxic side effects and limited efficacy, in addition to resistance [2]. Therefore, there is a dire need for searching for novel agents targeted against oesophageal cancer cells.

Plant-sourced bioactive molecules have provided architectural scaffolds for numerous lifesaving clinical agents, including 27% of the approved natural anticancer drugs, since 1980 [3]. Furthermore, over 3,000 global plant taxa have documented ethno-medicinal uses in the treatment of cancer [4].

Mauritius is a biodiversity hotspot located off the southeast coast of the African continent in the Indian Ocean. To date, the ethno-medicinal uses of about 32% of Mauritian endemic plants are documented with limited insight into their anticancer potential [5]. Therefore, this untapped unique resource represents a fertile ground for the bioprospecting of novel oncologic agents.

Thus, the present work evaluated the *in vitro *antioxidant and tumor cytotoxicity of *A. integrifolia*, *D. acutangula*, *E. tinifolia*, *G. psychotrioides*, and *L. glauca *Bojer (*Fig. 1*), in relation to their polyphenolic content. The effect of the three most potent extracts on cell cycle progression, mode of induced cell death, and their ability to modulate AMPK in oesophageal adenocarcinoma cells was also studied.

## METHODS

**Plant material and preparation of extracts**

Fresh leaves of *L. glauca*, *A. integrifolia, *and *G. psychotrioides *were collected at Gaulette serré, near Camp Thorel (coordinates 20° 12` 09`` S, 57° 25` 11`` E; 20° 12` 43`` S, 57° 38` 29`` E and 20° 12` 43`` S, 57° 38` 29`` E, respectively), while leaves of *E. tinifolia *and *D. acutangula *were collected at lower gorges national park, ‘Morne Sec’ (coordinates 20° 23` 35`` S, 57° 38` 05`` E) and Réduit (coordinates 20° 14` 05`` S, 57° 29` 45`` E), respectively. Dried leaves were exhaustively extracted with aqueous methanol (80 %, v/v) and freeze-dried as described [6].

**Estimation of polyphenolic contents**

The Folin-Ciocalteu assay, aluminum chloride assay, and HCl/Butan-1-ol assay were used to estimate the phenolic, flavonoid, and proanthocyanidin contents, respectively [6]. The results were expressed as mg of gallic acid equivalent (GAE)/g lyophilized weight (LW), quercetin equivalent (QE)/gLW, and cyanidin chloride equivalent (CCE)/gLW, respectively.

**Chromatographic determination of phenolic compounds**

The GC-MS analysis of trimethylsilylimidazole derivatized extracts of *L. glauca*, *E. tinifolia *and *A. integrifolia *was carried out using an Agilent 7890A gas chromatography system (Agilent Technologies, USA) as described in [7]. The analysis began with the initial oven temperature set at 150°C, which increased at the rate of 10°C/min to 300°C, and was maintained for another 4–5 min to yield a total run of 20 min under constant helium pressure (10 psi). The chromatogram was analyzed by matching the MS spectra of the peaks with those stored in the NIST 2011 Mass Spectral Library.

***In vitro *antioxidant capacities of extracts**

The *in vitro *antioxidant activities of the extracts were evaluated using ferric reducing antioxidant power (FRAP) assay; iron chelating assay; superoxide anion radical scavenging assay; and nitric oxide radical inhibition assay, as described in [6]. The modified method proposed by Chu et al. [8] was employed for the DPPH (2-2 diphenyl-1-picrylhydrazyl) radical scavenging activity. Briefly, 100 μl of a methanolic extract (of varying concentration) and 200 μl of 100 μM DPPH dissolved in methanol were incubated for 30 min at room temperature and the absorbance was read at 492 nm.

The negative and positive controls contained extract vehicle and gallic acid (or otherwise stated), respectively, instead of the extract. The percentage of metal chelating and free radical scavenging activity of the extracts were calculated with reference to the negative control according to equation 1. A concentration response curve was generated, and the IC_50_ value was determined using the GraphPad Prism 6 software (GraphPad Inc., USA). All the experiments were performed in triplicates in three independent assays.

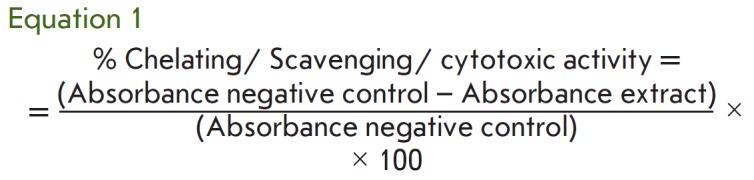



**Human cell cultures**

Human cancer cell lines purchased from American Type Culture Collection included cervical adenocarcinoma (HeLa), colorectal carcinoma (HCT 116), oesophageal adenocarcinoma (OE 33, FLO-1, OE 19 (platinum resistant)), and oesophageal squamous cell carcinoma (KYSE-30), while non-malignant ones included retinal pigment (RPE-1) and fibroblast (FIBR) cell lines. HeLa, HCT 116, FLO-1, RPE-1, and FIBR were grown in DMEM, while OE 33, OE 19, and KYSE-30 were cultured in RPMI-1640, both media supplemented with 10% (v/v) fetal calf serum (FCS), 2 mM *L*-glutamine, 1% Penicillin-Streptomycin solution with the medium for RPE-1 having an additional supplementation of 20% sodium carbonate. The cells were grown under standard culture conditions.

**Cell proliferation analysis using the metabolic assay**

The HeLa, HCT 116, FLO-1, OE 33, OE 19, KYSE-30, RPE-1, and FIBR cell lines were plated at 1 × 10^4^ cells per well in a 96-well plate and incubated for 24 h before treatment. The medium was replaced with a fresh medium containing the test extracts (0.78, 1.56, 3.13, 6.25, 12.5, 25 and 50 μg/ml). The experimental negative and positive controls included 0.1% (v/v, final concentration) dimethyl sulfoxide (DMSO) and etoposide (0.78, 1.56, 3.13, 6.25, 12.5 and 25 μg/ml), respectively. After 24 h, 10 μl of Alamar blue reagent (10% (v/v)) was added and the plates were incubated at 37°C for 4 h. Fluorescence was measured using an excitation wavelength of 544 nm and an emission wavelength of 590 nm in a Synergy H4 Hybrid Multi-Mode Microplate Reader (BioTek, USA). Cell viability of the treated cells was calculated as a percentage of the number of viable cells in the negative control (equation 1) and the *IC_50_*value determined. The results were expressed as the mean *IC_50_*± SD μg/ml (n=3). The selectivity index (SI) values for the test extracts were calculated as the ratio of *IC_50_*values of RPE-1 cells to cancer cells.

**Cell death assay and cell cycle analysis**

FLO-1 and KYSE-30 cells were seeded (2.6 × 10^4^ and 1.8 × 104, respectively) in 384-well Greiner, black, tissue culture plates. The plates were incubated for 24 h under standard culture conditions before test extracts (*L. glauca*, *E. tinifolia *and *A. integrifolia*) were added using a Biomek FX liquid handler (Beckman Coulter Inc., USA), to give 6-point dose responses with final assay concentrations of 30, 10, 3, 1, 0.3, 0.1 μg/ml with four replicates. DMSO and Staurosporine (3, 1, 0.3, 0.1, 0.03, 0.01 μM) were added as controls. The cells were further incubated for 48 h before staining. The media were aspirated from the plate wells and replaced with the staining mixture (Hoechst 33342 (2 μg/ml, Invitrogen) and MitoTracker Deep Red FM (500 nM, Invitrogen)). The plates were incubated in the dark (30 min), before washing three times with PBS. The staining solution was replaced with normal media, and the plates were imaged on an ImageXpress system (Molecular Devices, UK), taking four images per well. The percentage of cells in each cell cycle phase was determined using the cell cycle application module within the MetaXpress software (Molecular Devices, UK)

**Reverse phase protein array (RPPA)**

Samples were analyzed by Zeptosens RPPA as described previously [9]. Briefly, tumor lysates were normalized to 2 mg/ml with CLB1 lysis buffer and diluted 1:10 in CSBL1 spotting buffer (Zeptosens-Bayer) prior to preparing a final 4-fold concentration series of 0.2, 0.15, 0.1, and 0.75 mg/ml. The diluted concentration series of each sample was printed onto Zeptosens protein microarray chips (ZeptoChipTM, Zeptosens-Bayer) under environmentally controlled conditions (constant 50% humidity and 14°C) using a non-contact printer (Nanoplotter 2.1e, Gesim). A single 400 picoliter droplet of each lysate concentration was deposited onto the Zeptosens chip (thus representing four spots per single biological replicate). A reference grid of AlexaFluor647 conjugate BSA consisting of four column X 22 rows was spotted onto each subarray; each sample concentration series was spotted in between the reference columns. After array printing, the arrays were blocked with an aerosol of a BSA solution using a custom-designed nebulizer device (ZeptoFOGTM, Zeptosen-Bayer) for 1 h. The protein array chips were subsequently washed in double-distilled water and dried prior to performing the dual antibody immunoassay comprising 24-hour incubation of primary antibody, followed by 2.5-hour incubation with the secondary Alexa-Fluor conjugated antibody detection reagent (anti-rabbit A647 Fab).

A selected panel of antibodies, pre-validated for RPPA application, was used. Following secondary antibody incubation and final wash step in the BSA solution, the immune stained arrays were imaged using the ZeptoREADERTM instrument (Zeptosens-Bayer, Germany). For each subarray, five separate images were acquired using different exposure times ranging from 0.5-10 s. Microarray images representing the longest exposure without saturation of fluorescent signal detection were automatically selected for analysis using the ZeptoViewTM 3.1 software (Zeptosens-Bayer, Germany). An error weighted least squares linear fit through the fourfold concentration series was used to calculate the median relative fluorescence intensity (RFI) value for each sample replicate. Local normalization of the sample signal to the reference BSA grid was used to compensate for any intra- inter-array/chip variation. Local normalized RFI values were used for the subsequent analysis, and the data are presented as a fold change over DMSO control samples.

**Statistical analysis**

Statistical analysis was carried out using the GraphPad Prism 6 software. The mean values were compared using One-Way ANOVA. Student’s t-test and/or Tukey’s multiple comparisons as the Post Hoc test was used to determine the significances of the mean cytotoxic activities of phytochemicals and antioxidants among different extracts and the positive control. All charts were generated using the Microsoft Excel software (version 2010).

## RESULTS

**Phytochemical analysis**

**Fig. 2:**
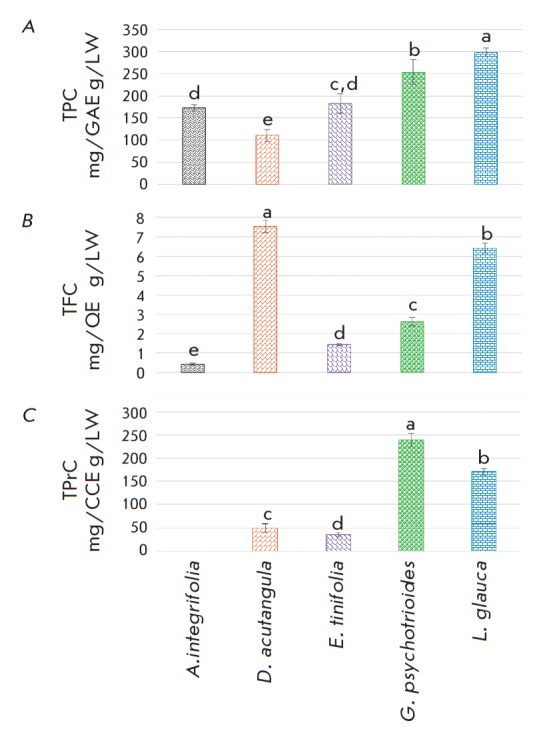
Total phenolic, flavonoid, and proanthocyanidin contents in the investigated extracts. LW – Lyophilized weight; CCE – cyanidin chloride equivalent; GAE – gallic acid equivalent; QE – quercetin equivalent; TPC – total phenolic content; TFC – total flavonoid content; TPrC – total proanthocyanidin content. The different superscripts between the columns represent a significant difference between the extracts (*p* < 0.05). The data are expressed as the mean ± standard deviation (*n *= 3)

**Fig. 3:**
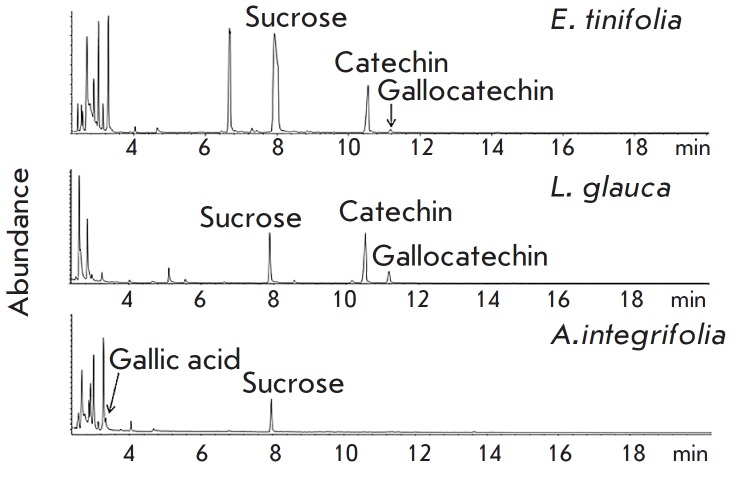
GC Chromatogram of TMSi derivatives of crude extracts

The TPC varied significantly between the investigated leaf extracts (p < 0.05), with the highest level measured in *L. glauca* (*Fig. 2*). The TPC in terms of gallic acid equivalent ranged from 298.9 ± 9.4 to 110.4 ±13.7 mg GAE/gLW. *G. psychotrioides *had the most abundant TPrC in terms of the cyanidin chloride equivalent, while a negligible amount was measured in the *A. integrifolia *leaf extract. The total flavonoid levels ranged between 7.6 mg ± 0.3 and 0.4 ± 0.1 mg QE/gLW, with *D. acutangula *having the most prominent flavonoid level followed by *L. glauca*. GC-MS analysis revealed the presence of (+)-catechin and gallocatechin in *E. tinifolia *and *L. glauca*, while gallic acid was detected only in *A. integrifolia* (*Fig. 3*).

**Antioxidant activities of extracts in *in vitro* models**

The trend in antioxidant potential between the extracts differed in each of the investigated assays (*Table 1*). The FRAP value of *L. glauca *was significantly higher than that of other endemic plant extracts (p < 0.05), and 2.4-fold lower compared to the positive control gallic acid (24.9 mmol Fe^2+^). All the extracts under study showed dose-dependent metal chelating and free radical scavenging activity; the *IC_50_*values are shown in *Table 1*. *L. glauca *had the most potent radical scavenging activity against DPPH and NO radicals. While *G. psychotriodes *had the highest O_2_.- radical scavenging activity, the metal ion chelating activities of *A. integrifolia *and *E. tinifolia *were notable compared to those of other investigated extracts.

**Table 1:** Antioxidant potential of the investigated medicinal plants leaf extracts

Extracts	Ferric reducing antioxidant power^1^	Iron chelating activity^2^	DPPH Scavenging activity^3^	Superoxide scavenging activity^3^	Nitric oxide scavenging activity^3^
*A. integrifolia*	5.8 ± 0.6d,^****^	655.7 ± 50.4a,^****^	9.4 ± 0.9c,^****^	9.4 ± 2.6b,^****^	528.4 ± 18.0b,^****^
*L. glauca*	12.1 ± 0.5a,^****^	6783.0 ± 1562.0c,^*^	2.2 ± 0.1a,^**^	7.6 ± 0.3a,b,^*^	10.5 ± 1.0a
*G. psychotrioides*	9.9 ± 0.7b,^****^	2189.0 ± 483.3b,^****^	2.8 ± 0.3a,^****^	6.4 ± 0.6a	21.7 ± 6.4a
*E. tinifolia*	8.4 ± 0.5c,^****^	674.1 ± 87.7a,^****^	4.4 ± 0.4b,^****^	8.9 ± 1.1b,^***^	15.6 ± 2.0a
Gallic acid	24.9 ± 0.9	8002.0 ± 169.6 (47.0 ± 1.0 mM)	0.7 ± 0.1 (3.9 ± 0.6 μM)	5.5 ± 0.2 (32.1 ± 1.3 μM)	9.9 ± 3.4 (58.4 ± 20.1 μM)

^1^1Values are expressed in mmol Fe^2+^;

^2^IC_50_ values are expressed in μg/ml;

^3^IC_50_ values are expressed in μg/ml;

Data represent a mean ± standard deviation (n = 3).

Different letters between rows in each column represent significant differences between the extracts (p < 0.05).

Asterisks represent significant differences between the extracts and gallic acid (positive control),

^*^p ≤ 0.05,

^**^p ≤ 0.01,

^***^p ≤ 0.001,

^****^p ≤ 0.0001.

**Cytotoxicity of herbal extracts**

The *A. integrifolia *and *E. tinifolia *extracts exhibited dose-dependent growth inhibition of the tested cancer cell lines. Both extracts were strongly cytotoxic towards KYSE-30 cells and were 6.9- and 5.6-fold, respectively, less toxic with respect to the immortalized normal RPE-1 cell line. The chemotherapeutic agent etoposide exhibited greater cytotoxicity toward all the tested cancer cell lines, relative to the extracts. However, it is worth noting that etoposide was more cytotoxic to wards RPE-1 (SI = 2.3) and fibroblast (SI = 1.5) cells compared to KYSE-30 cells. The calculated *IC*50 value for each extract against the tested cell lines is shown in *Table 2*.

**Table 2:** Cytotoxicity (IC_50_ μgLW/ml) of extracts against Human cell lines

Extracts	Flo-1	OE 33	OE 19	KYSE-30	HeLa	HCT 116	Fibroblast	RPE1
*A. integrifolia*	10.43 ± 2.10 [SI = 4.2]^c*^	28.03 ± 4.21 [SI = 1.6]^a****^	39.28 ± 7.52 [SI = 1.1]^a^	6.42 ± 2.21 [SI = 6.9]^a^	7.67 ± 1.58 [SI = 5.7]^a^	14.51 ± 1.23 [SI = 3.0]^b,c****^	36.93 ± 6.41 ^b****^	43.99 ± 4.76 ^a,b****^
*D. acutangula*	45.72 ± 7.93 [SI = 0.8]^a****^	ND	ND	ND	14.27 ± 2.97 [SI = 2.6]^b****^	14.13 ± 2.62 [SI = 2.7]^c****^	45.30 ± 4.33 ^a****^	37.47 ± 4.50 ^b****^
*E. tinifolia*	48.62 ± 3.24 [SI = 0.8]^a****^	45.72 ± 7.93 [SI = 0.9]^b****^	44.65 ± 8.87 [SI = 0.9]^b^	6.99 ± 0.38 [SI = 5.6]^a^	35.26 ± 5.01 [SI = 1.1]^c****^	19.54 ± 5.16 [SI = 2.0]^b****^	27.04 ± 1.84 ^c****^	38.94 ± 4.10 ^b****^
*L. glauca*	11.19 ± 2.53 [SI = 4.3]^c*^	ND	ND	9.22 ± 1.16 [SI = 5.2]^b**^	41.53 ± 3.28 [SI = 1.2]^d****^	31.58 ± 5.25 [SI = 1.5]^a****^	26.74 ± 1.64 ^c****^	48.49 ± 6.12 ^a****^
Etoposide	5.23 ± 0.56 [SI = 3.1]	6.74 ± 0.66 [SI = 2.4]	ND	7.05 ± 0.89 [SI = 2.3]	5.47 ± 0.66 [SI = 3.0]	4.93 ± 0.35 [SI = 3.3]	10.24 ± 2.77	16.17 ± 3.93

Data represent mean IC_50_ values ± standard deviation (n = 3).

Selectivity index determined as a ratio of the IC_50_ immortalized RPE-1 normal cell to the IC_50_ of cancer cell lines is indicated.

ND = at 50 and 25 μg/ml the extracts and etoposide, respectively, failed to induce 50% growth; hence, no IC_50_ value was determined.

Different letters between the rows in each column represent significant differences between the extracts (p < 0.05).

Asterisks represent significant differences between the extracts and etoposide (positive control),

^*^p ≤ 0.05,

^**^p ≤ 0.01,

^***^p ≤ 0.001,

^****^p ≤ 0.0001.

**Cell cycle analysis**

**Fig. 4:**
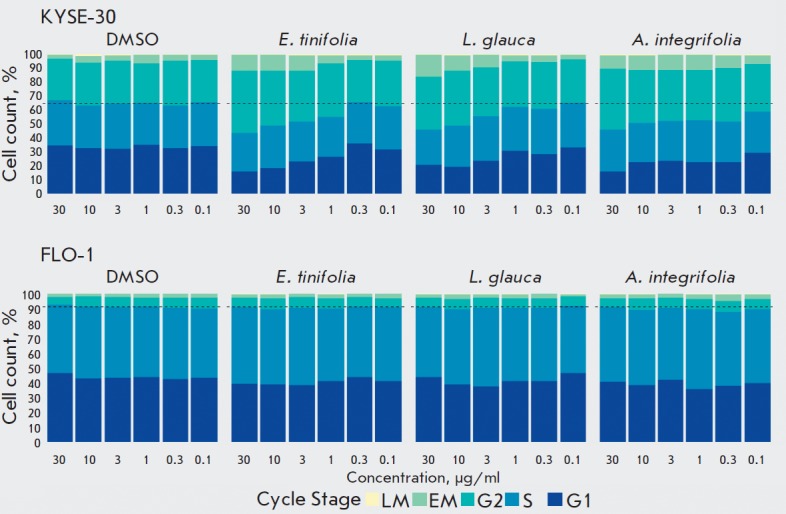
Cell cycle distribution after extract treatment in the KYSE-30 and FLO-1 cell lines. Cell cycle distribution was analyzed by high-content microscopy (ImageXpress-Micro). *E. tinifolia *and *L. glauca *caused significant G2/M arrest down to 3 μg/ml (Student’s *t*-test, *p * < 0.05), and the effect caused by *A. intergrifolia *was significant at all concentrations

The cell cycle stage of cells determined using the Cell Cycle Application Module within the MetaXpress software (Molecular Devices, UK) revealed that the extracts affected oesophageal squamous cell carcinoma (KYSE- 30) selectively, showing no changes in the oesophageal adenocarcinoma (FLO-1) cell line. Both the *E. tinifolia *and *L. glauca *extracts exhibited a concentration-dependent effect on the cell cycle (*Fig. 4*), causing significant G2/M arrest of KYSE-30 cells, down to 3 μg/ml (p < 0.05). Even though the *A. integrifolia* extract also caused cell cycle arrest at G2/M, the effect was less concentration-dependent. None of the extracts caused any changes in the cell cycle of FLO-1 cells (*Fig. 4*).

**Fig. 5:**
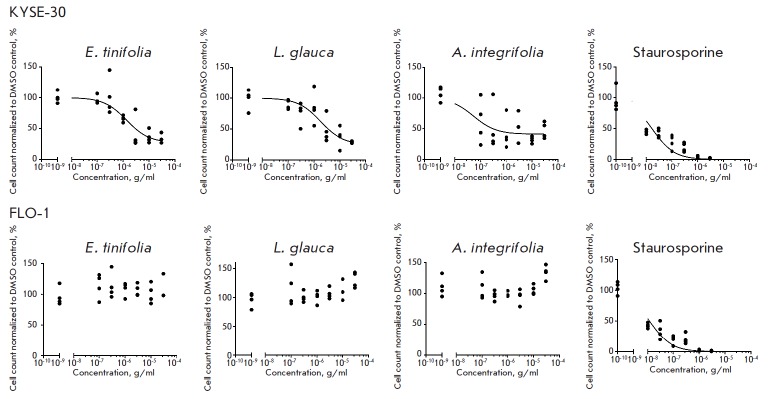
Concentration response graphs for the extracts and Staurosporine control cytotoxicity on the KYSE-30 and FLO- 1 cell lines. The DMSO controls to which the data were normalized were included for reference (data points left of the break on the x-axis)

Interestingly, a similar selectivity pattern was observed in the cell death assays. The number of nuclei per image was determined using the MetaXpress software. Extracts of *E. tinifolia *and *L. glauca *induced cell death in KYSE-30 cells in a concentration-dependent manner. However, both extracts had no effect on FLO-1 cells. The calculated *IC_50_*for *E. tinifolia *against KYSE-30 was 1.37 μg/ml; *IC_50_*for *L. glauca was *1.77 μg/ml. *E. tinifolia *at a concentration of 1 μg/ml significantly decreased the KYSE-30 cell number relative to the negative control (*p * < 0.05), while for *L. glauca *a significant reduction in the cell number was observed at 3 μg/ml (*p * < 0.05) (*Fig. 5*).

**Fig. 6:**
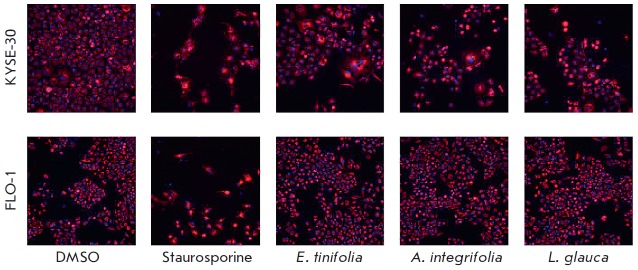
Fluorescence microscopy images showing cell number and morphological changes in the KYSE-30 and FLO-1 cells. The cells were treated with the extracts (3 μg/ml) and Staurosporine (0.3 μM) for 48 h

The selective effect on KYSE-30 was based on the effects of the extracts on cell morphology (*Fig. 6*). The three extracts had a marked effect on the cell number, cell and nuclear morphology, being indicative of cytotoxic and cytokinesis defects. However, there were no apparent morphological changes in the FLO-1 cells (*Fig. 6*).

**RPPA results**

**Fig. 7:**
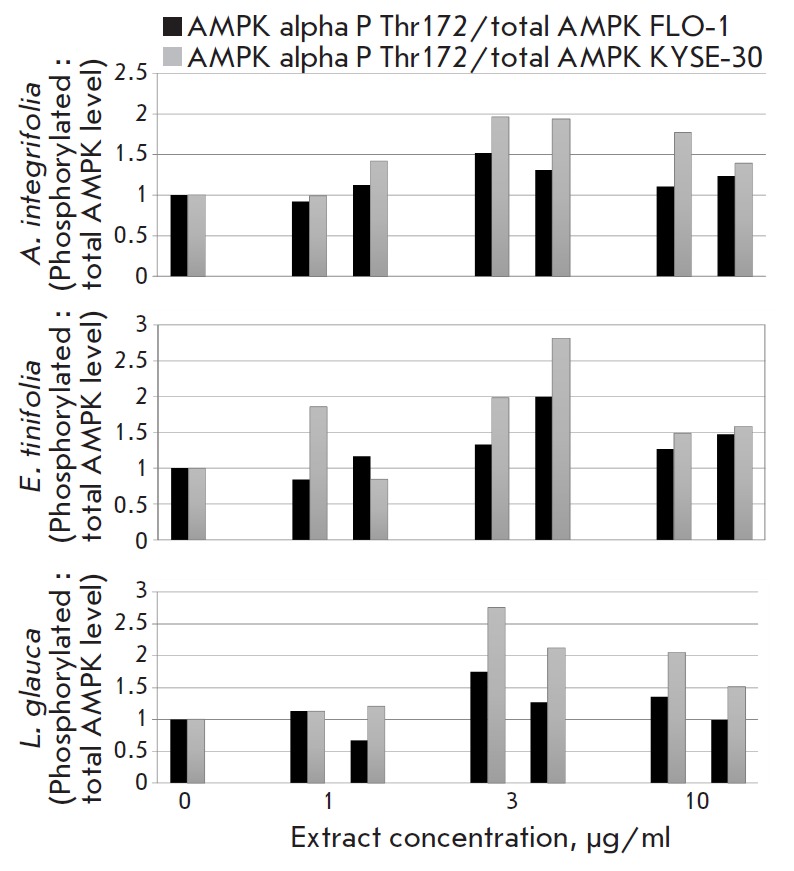
Graphs showing the changes in the activated phosphorylated AMPK level in the FLO-1 and KYSE-30 cell lines. The cells were treated for 3 h at different concentrations. The results represent the ratio of phosphorylated to total AMPK levels and are expressed as a fold change over DMSO control for each cell type

A selected panel of antibodies, pre-validated for RPPA application, was used to compare the individual levels of the corresponding proteins in cancer cells treated with each of the extracts (at different concentrations) with the mock-treated cells. The comparison of the RPPA analysis revealed that, at the 3-h time point, all three extracts upregulated the level of Thr-172 phosphorylation of the alpha subunit of AMPK in a dose-dependent manner, peaking at 3 μg/ml (*Fig. 7*), suggesting the AMPK pathway of activation in cancer cells.

## DISCUSSION

The role played by the chemical scaffolds provided by terrestrial plants for lifesaving drugs is immense [3]. To date, only about 15% of the global plant species have been appraised for their curative potential [10]. In Mauritius, about 32% of the endemic flora have ethnomedicinal uses, although there has been limited scientific validation of their efficacies [5]. The majority of the untapped endemic species, both medicinal and non-medicinal, continue to represent a valuable source of novel chemotypes that await discovery. The therapeutic benefits of plants are ascribed to their secondary metabolites, which are broadly grouped into alkaloids, terpenoids, and phenolics, of which the latter represent an interesting class [11].

A high level of phenolics was estimated in *L. glauca*, *G. psychotriodes*, *E. tinifolia, *and *A. integrifolia*. *D. acutangula *had the highest flavonoid level, although a low total phenolic content was measured, closely followed by *L. glauca*. The polyphenolic richness of Mauritian plant extracts has been extensively reported on and may be attributed to the high sunlight conditions of the island [12]. Furthermore, a number of reports have delineated the phenolic composition of selected Mauritian endemic plant leaf extracts [13-17]. Consistent with the current findings, previous reports by Neergheen and co-workers demonstrated the relatively higher abundance of total proanthocyanidin compared to total flavonoid components in a *G. psychotriodes *leaf extract [15, 18]. Moreover, the occurrence of flavan-3-ols, namely, (+)-catechin and (-)-epigallocatechin, detected in the *E. tinifolia *leaf extract (*Fig. 3*) is concordant with findings in the literature [18]. Polyphenolic compounds, inclusive of flavan-3-ols derivatives, are extensively documented with regard to their antioxidant capacities [11, 19].

The pivotal role of plant polyphenols in modulating intracellular redox homeostasis and mitigating oxidative stress-induced pathologies is well established [19]. Mechanistically, antioxidants may neutralize free radicals either by single electron or hydrogen atom transfer [20]. The antioxidant capacity of phenolic compounds emanates from the presence and degree of an electron-donating hydroxyl group on the aromatic ring [21]. Polyphenols, for instance flavonoids, are multifunctional in their antioxidative ability and prevent oxidative damage in multiple ways, including free radical scavenging through direct donation of hydrogen atoms, inactivating enzymes due to hydrogen bonding of hydroxyl groups to proteins and chelating of the metal iron involved in free radical generation, among others [22]. Given the diverse mode of action of phenolic antioxidants, the present study employed a battery of five independent *in vitro *models to determine the antioxidant potential of the extracts. *L. glauca *exhibited the most effective antioxidant capacity in terms of ferric-reducing potential and scavenging of DPPH and nitric oxide free radicals. The antioxidant capacity of the endemic leaf extracts correlated significantly with the total phenolic content r = -0.887 (p < 0.05) for DPPH free radical scavenging activity and r = 0.970 (p < 0.01) for FRAP. The strong correlation between TPC and FRAP may be partly attributed to the fact that both assays share a similar redox mechanism [23]. Similar linear correlation between the TPC and FRAP values of Mauritian endemic leaf extracts was previously reported [17]. The positive association between the phenolics and antioxidant activity is not restricted only to Mauritian endemic plants, as comparable relationships have also been reported for Mauritian citrus fruits [24] and Mauritian tea infusate extracts [14].

Given the involvement of oxidative stress in the multistage carcinogenic process [25], polyphenol-rich extracts are expected to prevent or halt the progression of cancerous cell growth. Polyphenolics are reported to attenuate *in vitro *cancerous cell growth via diverse mechanisms [26]. The *in vitro *antiproliferative potential of selected Mauritian endemic *Eugenia *and *Syzygium *species against breast cancer cells (MDA-MB and MCF-7) has been described earlier [27]. However, this study reports for the first time on the growth inhibitory activity of Mauritian endemic leaf extracts against cervical adenocarcinoma, colorectal carcinoma, and oesophageal squamous cell carcinoma. The leaf extracts of *A. integrifolia*, *L. glauca, *and *E. tinifolia *exhibited dose-dependent growth inhibition against the selected cancer cell lines (*Table 2*). However, oesophageal squamous cell carcinoma KYSE-30 cells were the most sensitive to the extracts’ treatment. As per the United States National Cancer Institute cytotoxicity guidelines, crude extracts having an *IC_50_*value below 20 μg/ml are considered active against tested cell lines [28]. In the present study, the calculated *IC_50_*values ranged between 6.42 and 9.89 μg/ml when KYSE-30 cells were co-cultured with *A. integrifolia*, *L. glauca, *and *E. tinifolia *extracts for 24 h. Furthermore, the selective cytotoxicity of the extracts toward KYSE-30 cells was fivefold or greater compared to immortalized non-malignant normal cells.

Guided by the AlamarBlue™ assay results, the effects of the three extracts on cell death and cell cycle stages of KYSE-30 and FLO-1 cells were further investigated across a concentration dilution series using fluorescence staining and high-content image analysis. The findings indicated that extract treatment for 48 h induces considerable dose-dependent cell death in KYSE-30 cells but not in FLO-1 cells (*Fig. 5*, *Fig. 6*). A similar trend was also observed in cell cycle analysis, as extracts treatment induced accumulation of KYSE- 30 cells in the G2/M phase, but no effect on the FLO-1 cell cycle was apparent (*Fig. 4*). The blockade of KYSE- 30 cells through G2/M was concentration-dependent for both *E. tinifolia* and *L. glauca*, while in the case of *A. integrifolia *the effect was less influenced by the extract dose.

In cancerous cells, signaling components of different cellular pathways are often mutated [29]. In this vein, activation of AMPK and subsequent inhibition of the mTOR pathway is an area of active research [30]. It was previously suggested that activators of AMPK, which include plant polyphenols, can interfere with tumor growth via cell cycle arrest [31] and apoptosis [32, 33] in cancerous cells. The RPPA analysis of the canonical signaling pathways that regulate the cell cycle progression and survival pathways, which in turn regulate cell cycle progression and survival, revealed that all three extracts increase the level of Thr-172 phosphorylation of the alpha subunit of AMPK in KYSE-30 cells (*Fig. 7*), suggesting its activation. This study showed for the first time that the antiproliferative effect of Mauritian endemic medicinal plant leaf extracts is related to their influence on the AMPK pathway modulation.

## CONCLUSIONS

In conclusion, the specific selectivity of *A. integrifolia*, *E. tinifolia, *and *L. glauca *to KYSE-30 oesophageal squamous cell carcinoma relative to oesophageal adenocarcinoma (FLO-1) calls for further investigation into the anticancer effect of these medicinal plants. This is further substantiated by the G2/M phase cell cycle arrest, leading to cell death. AMPK pathway modulation following the exposure of KYSE-30 cells to the extracts provides novel insight into the mechanism of action of these leaf extracts as a potential chemotherapeutic treatment. This is particularly important since esophageal cancer is among the leading causes of cancer mortality worldwide as treatment is limited due to adverse systemic effects, limited efficacy, and emergence of drug resistance. Clinical studies with molecularly targeted therapies have so far been disappointing, with little improvement in patient outcomes. Hence, there is an urgent need to search for new effective treatments for oesophageal cancer. Although preliminary characterization indicated the presence of (+)-catechin and gallocatechin, in-depth phytochemical identification is warranted in order to identify the AMPK modulator.

## CONFLICT OF INTEREST

The authors declare the lack of any conflict of interest.

## Abbreviations

AMPK: 5′AMP-activated kinase
CCE: cyanidin chloride equivalent
DPPH: 2-2 diphenyl-1-picrylhydrazyl
EDTA: ethylenediaminetetraacetic acid
FRAP: ferric reducing antioxidant power
GAE: gallic acid equivalent
GC-MS: gas chromatography coupled to mass spectrometry
HPLC: high-performance liquid chromatography
LW: lyophilized weight
mTORC1: mammalian target of rapamycin complex 1
OC: oesophageal cancer
QE: quercetin equivalent
RFI: relative fluorescence intensity
RPPA: reverse phase protein array
TFC: total flavonoid content
TMSi: trimethylsilylimidazole
TPC: total phenolic content
TPrC: total proanthocyanidin content
